# Bone Tumours of the Talus: 18-Year Cohort of Patients With Rare Osteoid Lesions

**DOI:** 10.7759/cureus.13565

**Published:** 2021-02-26

**Authors:** Luke F Western, Rohit Dhawan, Gillian Cribb, Karen Shepherd, Paul Cool

**Affiliations:** 1 Orthopaedic Surgery, Oxford University Hospitals NHS Foundation Trust, Oxford, GBR; 2 Arthroplasty, Robert Jones and Agnes Hunt Orthopaedic Hospital NHS Foundation Trust, Oswestry, GBR; 3 Orthopaedic Oncology, Robert Jones and Agnes Hunt Orthopaedic Hospital NHS Foundation Trust, Oswestry, GBR; 4 Medical Sciences, Keele University, Keele, GBR

**Keywords:** hindfoot, talus tumour, osteoid osteoma, osteoblastoma, chondroblastoma

## Abstract

Background

Bone tumours of the talus are a rare cause of ankle pain. This study aims to provide additional clinical clarity regarding the presentation and management of a minimally researched topic.

Methods

Sixteen patients were diagnosed with bone tumour of the talus between 2002 and 2020 following referral for ankle pain. Symptoms, diagnosis, and management were retrospectively reviewed. Patients were actively followed up until consistently symptom-free and consenting to discharge (mean of 2.9 years). An open appointment was offered to all patients to reattend the unit if symptoms recurred.

Results

The most common diagnosis was osteoid osteoma/osteoblastoma (nine patients), chondroblastoma (four patients), a giant cell tumour of bone, a chondral lesion in Ollier’s disease and a rare metastatic renal cancer case. The mean age of onset was 29 years. Thirteen patients experienced ankle pain without a clear precipitating cause. Night pain was less common in osteoid osteoma/osteoblastoma than usually observed in the literature. The mean delay in diagnosis was two years, often due to an incorrect diagnosis of soft tissue injury. Plain radiographs are insufficient to identify most lesions.

Ten patients underwent computed tomography (CT)-guided radiofrequency ablation and five patients had open surgical curettage. Ollier’s disease was managed with orthotics. The five cases of recurrence across four patients were managed operatively.

Conclusions

Patients are usually young and healthy with benign disease, but talus tumours can cause significant functional impairment. Unexplained ankle pain should be extensively examined and be further investigated with magnetic resonance imaging (MRI) and CT scanning to avoid missing these rare tumours.

## Introduction

Bone tumours of the foot are a rare cause of ankle pain. It is estimated that only 3% of osseous tumours occur within the foot. Of those tumours, only 8% are within the talus bone [1-2]. However, focused literature regarding talus tumours is uncommon and limited, with talus specific data contained only within three papers [3-5]. Descriptions of talus tumours are otherwise contained within case series describing various bones of the feet [2,6-7], tumour-specific reviews [4,8-9] or single case reports [10-13]. Further demonstration of these rare tumours may help improve insight into the specific behaviour of talus tumours, gain a greater understanding of outcomes and promote diagnostic efficiency.

This study aims to highlight the clinical presentation and management of talus bone tumours.

## Materials and methods

A retrospective cohort of patients with talar lesions referred to our unit between 2002 and 2020 was identified for this study. Full electronic case records were reviewed by two authors independently. All patients with neoplasms of the talus bone were included in this study. Non-neoplastic lesions or soft tissue tumours invading the talus were excluded.

Patient demographics, details of the presentation, presenting symptoms, diagnostic details, management and complications were collected for analysis. All data were anonymised before analysis.

All cases were discussed at the multi-disciplinary team meeting where the diagnosis was confirmed, and best practice management discussed. With consultation of the risks and benefits with the patient, a management plan was agreed upon. Tumours were classified according to the World Health Organisation classification [14]. Although histologically similar to osteoblastoma [11,15-16], osteoid osteomas are smaller than 20mm, whilst osteoblastomas are larger than this size [14]. All diagnoses were confirmed histologically. The date of histological diagnosis was used as the date of definitive diagnosis.

The tumour size was measured from fat-sensitive magnetic resonance imaging (MRI) as the largest cross-sectional diameter. Initial treatment for benign talus tumours often utilises computed tomography (CT)-guided radiofrequency ablation or surgical curettage without bone grafting. CT-guided radiofrequency ablation was performed using multi-tinned expandable electrodes as described previously [17-19].

Patients were followed up until they were consistently symptom-free with normal function and discharged with an open appointment to attend in case of recurrence of symptoms. Any recurrence was identified, and subsequent management recorded. From 2015, the Toronto Extremity Salvage Score (TESS) was used as part of the standard postoperative functional assessment and included in the evaluation.

## Results

Twenty-one patients with lesions of the talus were identified over eighteen years. Five patients with non-tumour or soft tissue lesions were excluded from the analysis as described; Simple bone cyst (n=2), geode (n=1) and tenosynovial giant cell tumours with invasion into the talus bone (n=2). Sixteen patients were confirmed with a bone tumour of the talus. The mean age at onset of symptoms was 29 years (range 14 - 53). Gender differential included nine male and seven female patients. The right talus was affected in eleven patients and the left in five patients. The mean follow-up was 2.9 years. There were fifteen benign tumours and one patient had metastatic renal cancer. Seven patients had an osteoid osteoma and two an osteoblastoma. There were four chondroblastomas, one giant cell tumour of bone, one chondral lesion in a patient with Ollier’s disease and one patient had metastatic renal cancer. Individual patient information is detailed in Table 1. 

**Table 1:** Individual cases Sixteen patients with bone tumours of the talus. Unexplained implies there was no obvious predisposing injury or other cause of pain. Prior management describes any management the patient received prior to referral and subsequently the reason for delay. Time to specialist review was time from date of first symptom presentation to date first seen at the specialist unit. TESS, a post-operative outcome measure is included for patients seen after 2015. Key: M (male), F (female), UAP (unexplained ankle pain), NAP (unexplained nocturnal ankle pain), AP (ankle pain), CT RFA (computed tomography-guided radiofrequency ablation), TESS (Toronto Extremity Salvage Score)

Patient	Diagnosis	Lesion Size (mm)	Side	Age of onset (years)	Sex	Presenting Complaint	Prior Diagnosis	Time to specialist review (months)	Procedure	TESS	Complication (time interval months)	Time to discharge (months)
1	Chondroblastoma	24	Right	33	M	UAP	Soft tissue	12	CT RFA	60	-	26.5
2	Chondroblastoma	33	Right	14	F	UAP	Tendinitis	24	Curettage	90	-	118.3
3	Chondroblastoma	34	Left	20	M	UAP	Soft tissue	32	CT RFA	82	-	9.2
4	Chondroblastoma	18	Right	23	F	UAP	None	8.8	Curettage	75	-	-
5	Osteoblastoma	23	Right	15	M	NAP	None	3.7	CT RFA		Recurrence (7.3)	124.4
6	Osteoblastoma	20	Right	32	M	AP (football injury)	Soft tissue	13.7	Curettage	79	Recurrence (7.8)	44.4
7	Osteoid Osteoma	16	Right	26	M	UAP	Osteochondral	36	CT RFA	51	-	21
8	Osteoid Osteoma	15	Left	34	F	UAP	None	11.7	Curettage		Recurrence (29.7)	32.9
9	Osteoid Osteoma	7	Right	50	M	NAP	Soft tissue	36	CT RFA		-	3.2
10	Osteoid Osteoma	6	Right	34	F	UAP	None	24	CT RFA	70	Mild heel pain (48)	23.6
11	Osteoid Osteoma	4	Right	30	M	NAP	Soft tissue	42.7	CT RFA	88	-	1.7
12	Osteoid Osteoma	5	Right	19	M	NAP	Soft tissue	12	CT RFA		-	3.7
13	Osteoid Osteoma	6	Left	35	F	NAP	Psoriatic arthritis	37.1	CT RFA		-	1.9
14	Giant Cell Tumour of Bone	40	Left	18	F	AP (curb injury)	Soft tissue	11.2	Curettage + Denosumab	96	Recurrence (31.2) Then Recurrence (12)	-
15	Chondral Lesion in Ollier’s Disease	25	Right	28	F	UAP, swelling	None	11.2	Orthotics	96	-	-
16	Metastatic Renal Cancer	28	Left	53	M	UAP	No delay	0.4	Curettage + cement		-	Died of disease at 12 months

All 16 patients presented with ankle pain. Thirteen patients had no clear possible precipitating factor, such as ankle trauma. The patient with Ollier’s disease also complained of associated swelling. Only four patients with osteoid osteoma and one patient with osteoblastoma complained of typical night pain. The remaining nine patients experienced ankle pain throughout the day. Only two patients had ankle pain that could potentially be attributed to a soft tissue injury (Table 1).

**Figure 1:**
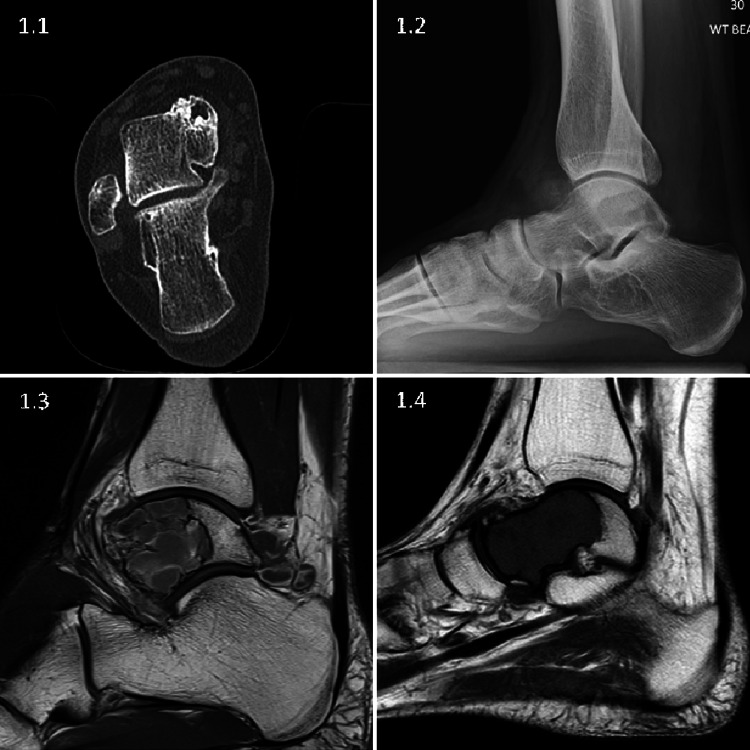
Talar bone tumours clinical images 1.1: Coronal CT scan of an osteoid osteoma demonstrating the nidus and the additional value of CT. 1.2: Lateral radiograph of an osteoblastoma, demonstrating difficulty to identify these lesions on plain radiography. 1.3: Sagittal section MRI scan of a chondroblastoma. 1.4: Sagittal section MRI scan of giant cell.

The mean delay in presentation to the specialist unit was 1.8 years (range 11 days to 3.6 years). A definitive histological diagnosis was made at a mean of two years (range 0.1 to 3.6 years) from the onset of symptoms. Only one patient was referred without delay; this patient had known renal cell carcinoma and presented with severe hindfoot pain and a large lesion on plain radiographs. Eight patients were incorrectly diagnosed with a ‘soft tissue injury’, one with an osteochondral injury and one with psoriatic arthritis, prior to referral. Five patients had no clear diagnosis recorded before referral. Patients had ongoing symptoms during the diagnostic delay, eventually leading to their referral. Specific care pathways in the community were not explored; Some patients had received radiographs at referral, but no further investigations were performed that could have highlighted the diagnosis before referral. At the initial presentation to our unit, all patients underwent further investigation: plain radiographs and MRI scans of their ankle. Our institution uses MRI, as it tends to identify soft tissue lesions more readily, as well as osteoid lesions, thus it is a good initial screening tool. Images can subsequently be used for accurate size calculation as described. CT was utilised when appropriate, particularly in suspected osteoid osteoma, osteoblastoma or chondroblastoma (Figure 1).

Nine patients underwent CT-guided radiofrequency ablation, and five patients underwent open surgical curettage without bone grafting. The curettage and cementation technique (poly methyl-methacrylate bone cement) was performed for the patient with renal cell carcinoma metastasis. The patient with a giant cell tumour of bone had received neo-adjuvant denosumab treatment to manage the tumour and aid operative technique in preparation for extensive curettage. The patient with Ollier’s disease was managed successfully with orthotics and continues to be followed up.

In total, four patients had a recurrence of disease at a mean of 1.7 years following diagnosis (two osteoblastomas, one osteoid osteoma and one giant cell tumour of bone). The patient with a giant cell tumour of bone has had two recurrent tumours. Three patients initially had open surgical curettage and one patient underwent CT-guided radiofrequency ablation. Two of the four recurrences were treated with CT-guided radiofrequency ablation (both patients had initial open surgical curettage). The other three recurrences were treated with open surgical curettage. One patient with chondroblastoma developed a stress fracture of the medial malleolus after excessive exercise four years following treatment. This was felt to be unrelated to the initial diagnosis, and the fracture healed following conservative management.

The patient with metastatic renal cell cancer died of the disease one year following treatment. He was walking unaided and had good function up to his death, with no clinical evidence of residual or recurrent talar tumour. Twelve patients have been discharged following successful treatment and are pain-free with normal function. Three patients remain under clinical surveillance. Another has a chondral lesion in Ollier’s disease, which necessitates ongoing follow-up. One patient is pain-free but remains under surveillance following recent diagnosis and treatment. The other has had a recent recurrence.

## Discussion

Bone tumours of the talus are rare, with only 16 patients being referred to this specialist orthopaedic hospital in 18 years.

The diagnoses of such tumours are understandably difficult in the community. Ankle pain with a possible precipitating cause, such as trauma, has a wide range of differential diagnoses to explore. Nevertheless, these patients, generally, had no clear precipitating cause for pain. Differentials in such cases may include cysts, gout, systemic arthritis (such as rheumatoid, lupus and psoriatic), stress fractures and non-traumatic ligamental injuries. However, with no further radiological, clinical or biochemical evidence, active clinical suspicion should be maintained, and rare causes, such as soft tissue and osteoid tumours, should be considered.

The majority of patients were young (less than 30) and healthy, and all but one patient had a benign diagnosis. This is consistent with current literature [1,3,20]. Though, despite benign pathology, patients experience persistent ankle pain that interferes with function and sleep. This is compounded by the delay in diagnosis (mean of two years). Tumours were of small size and radiological diagnosis via plain radiograph was challenging. These are likely important factors in the delay of referral to a specialist unit [14,21-23].

The common diagnosis in this cohort was osteoid osteoma/osteoblastoma. Apart from size, there is minimal histological difference between the two entities [11,14]. However, osteoblastomas tend to behave more aggressively and have a higher reported recurrence rate [14]. In this cohort, there were only two lesions with a maximal dimension larger than 20 mm that could be classified as osteoblastoma. Both lesions recurred. One osteoid osteoma recurred, which was 15 mm in size and the second-largest osteoid osteoma in the cohort. None of the smaller osteoid osteomas recurred. These findings are in keeping with the published literature [3-5,24]. Osteoid osteomas are readily identifiable on CT (Figure 1), and this imaging modality should be used to improve diagnosis and treatment planning [21].

Only five patients with an osteoid osteoma or osteoblastoma presented with pain at night, as typically described in the literature [14]. Furthermore, symptoms may respond to anti-inflammatories, but the response is often not as dramatic as is classically described in other osteoid osteoma sites [14,21,25]. A potential explanation of these observations is that night pain is caused by a local inflammatory reaction of the periosteum (periostitis) and associated reactive bone formation. However, as the talus is an intercalated bone, there is no periosteum, and the local inflammatory reaction evokes synovitis instead of periostitis. Consequently, the patients present with unexplained mono-arthritis. This deviation from the classical presentation may, in part, also explain the diagnostic delay.

Chondroblastoma usually presents as a small, lucent, periarticular bone lesion, making it particularly difficult to diagnose [14,22]. The knee is the most common site, but the tumour has been described in the talus. Although some matrix calcification exists, this is often extremely subtle and only visible on CT scans. Consequently, CT scans are recommended for further evaluation of suspected chondroblastoma [14,20,26]. Historically, extensive curettage has been favoured [24]. However, the efficacy of CT-guided radiofrequency ablation in the management of chondroblastoma has been demonstrated [17-19]. Two patients with chondroblastoma underwent CT-guided radiofrequency ablation and none of the four patients with chondroblastoma developed a recurrence.

A giant cell tumour of the bone is an aggressive benign bone tumour that can predispose patients to pathological fractures. The tumour is usually visible on plain radiograph and MRI scans. The management of giant cell tumour of bone is difficult, with a high risk of local recurrence [27-29]. denosumab has been used as a neo-adjuvant therapy to down-regulate the tumours [29-30]. The patient in this cohort received pre-operative denosumab. Despite the intervention, recurrence occurred 2.6 years following initial treatment and a second time one year following that.

The small size and subtle radiological features explain the delay in diagnosis in osteoid osteoma, osteoblastoma and chondroblastoma. The patients with giant cell tumour of bone and chondral lesion in Ollier’s disease had mild symptoms, explaining their diagnostic delay. The only patient who did not have a delay in diagnosis was the patient with malignant disease. He presented with severe symptoms, a known background of malignant renal cancer and a lesion that was readily identifiable on plain radiographs.

The decision to perform CT-guided radiofrequency ablation or open surgical curettage was taken with the patient following discussion at the multi-disciplinary team meeting. The intra-operative identification of small lesions can be challenging, and the majority of small lesions had radiofrequency ablation under CT guidance. Larger, more aggressive lesions usually had open curettage and these lesions were also the most likely to recur (three of the four recurrent tumours were following open surgical curettage). However, whether recurrence is related to the initial size of the lesion or the performed treatment can only be speculated.

Postoperative TESS showed good functional outcome in the majority of patients, with a mean average of 79% (Table 1). However, TESS is designed to assess lower limb function following limb salvage in sarcoma surgery and is not specifically validated for talar tumours. Furthermore, scores were not available for all patients (Table 1).

Long-term data sets are not available, and it is conceivable that radiofrequency ablation could damage articular cartilage and facilitate the onset of osteoarthritis. Given many of the lesions were small, the likelihood of this will hopefully be attenuated. All patients that were discharged received an open appointment to attend in case of further symptoms. None have yet re-attended.

Another limitation of our study is the potential selection bias due to the specialist nature of our practice.

We have identified a significant delay in diagnosis for this cohort of patients. The topic could be further researched by exploring possible contributing factors in the delay in diagnosis.

## Conclusions

Primary bone tumours of the talus are rare. Patients are usually young and although pathology is usually benign, a delay in diagnosis can leave patients with protracted pain, which impacts their function. Typical night pain, as reported for osteoid osteomas and osteoblastomas, is usually absent and patients present with symptoms of synovitis in the ankle joint. An MRI scan is recommended for further evaluation in patients with unexplained ankle pain, even in the presence of normal radiographs. A CT scan can help further characterise lesions and aid treatment planning.
